# Extracorporeal shock wave therapy for post-stroke spasticity: an umbrella review of systematic reviews and meta-analyses

**DOI:** 10.3389/fneur.2026.1705104

**Published:** 2026-04-20

**Authors:** Jiayi Chang, Yuwei Sun, Jiayi Zeng, Huini Gao, Yanlan Yu

**Affiliations:** 1 School of Nursing, Hunan University of Chinese Medicine, Changsha, China; 2 Department of Neurology, The First Affiliated Hospital of Hunan University of Chinese Medicine, Changsha, China

**Keywords:** extracorporeal shockwave therapy, meta-analyses, muscle spasticity, scientific rigor, stroke, umbrella review

## Abstract

**Introduction:**

This umbrella review aims to synthesize and critically evaluate existing systematic reviews and meta-analyses to determine the efficacy of extracorporeal shock wave therapy for post-stroke spasticity, along with the quality and reliability of the evidence.

**Methods:**

A comprehensive search of eight databases (up to May 2025) was conducted to identify systematic reviews and meta-analyses evaluating extracorporeal shock wave therapy for post-stroke spasticity. Two scholars independently screened the literature and extracted data. The methodological quality of the included systematic reviews and meta-analyses was appraised using AMSTAR 2, and the certainty of evidence for each outcome was rated using the GRADE approach. Overlap of primary studies across reviews was assessed and visualized using the GROOVE tool.

**Results:**

A total of 17 systematic reviews and meta-analyses were included. Among these, 3 were rated as high quality, 2 as moderate quality, 7 as low quality, and 5 as very low quality. Evidence mapping identified 136 nodes, indicating moderate overlap. All included systematic reviews and meta-analyses suggested that extracorporeal shock wave therapy improves post-stroke spasticity. Extracorporeal shock wave therapy helps reduce spasticity, improve sensorimotor function, increase active and passive range of motion, and alleviate pain.

**Discussion:**

This umbrella review suggests that extracorporeal shock wave therapy can improve post-stroke spasticity symptoms. However, due to the generally low methodological quality of the included systematic reviews and meta-analyses, the existing evidence remains limited and inconclusive. Language restrictions and the predominance of studies from a single country may also limit their generalizability. Further high-quality research is needed to strengthen the evidence base.

**Systematic review registration:**

identifier: CRD420251124065.

## 1 Introduction

Stroke is the second leading cause of death globally and the third leading cause of disability and death (1). By 2050, global stroke-related deaths are projected to increase by 50%, with disability-adjusted life years (DALYs) rising by 31% to 189.3 million, highlighting the immense global burden of stroke (2). Post-stroke spasticity (PSS) is a motor and sensory disorder characterized by a velocity-dependent increase in the muscle response to stretch, accompanied by exaggerated tendon reflexes resulting from hyperexcitability of the stretch reflex (3). Typically, it first appears within 2 weeks to 3 months after stroke onset, with an incidence rate of approximately 25%−43% (4). The onset of PSS spans a broad timeframe, typically emerging between 1 and 6 weeks and peaking within 1–3 months. Over time, especially during the chronic phase, the burden of PSS may progressively increase. Particularly among stroke survivors with moderate to severe motor deficits, spasticity prevalence can reach up to 97% (5). This phenomenon indicates that PSS persists not only during the acute and subacute phases but may evolve throughout the entire stroke course. Although functional outcomes can improve with appropriate chronic-phase interventions, PSS interacts with abnormal neuroplasticity to impede genuine motor recovery (6). The treatment options for PSS include BoNT injections, passive exercise and stretching, direct current stimulation, transcutaneous electrical nerve stimulation, vibration therapy, ultrasound therapy, acupuncture, orthotics, transcranial magnetic stimulation, thermotherapy, and cryotherapy (7). Among these, BoNT injections are widely recommended as first-line therapy and are considered one of the most effective interventions for spasticity (8). However, risks such as localized weakness post-injection and the development of neutralizing antibodies with repeated injections persist (9). ESWT, a non-invasive treatment utilizing high-energy pulsed sound waves, is regarded as a non-surgical alternative for spasticity management (10). Extracorporeal shock wave therapy (ESWT) improves spasticity symptoms and promotes functional recovery by applying mechanical stimulation to target tissues, thereby modulating neuromuscular excitability and tissue rheological properties (11). ESWT is categorized into focused and radial types. fESWT delivers concentrated energy to deep targets with strong penetration, making it suitable for treating deep muscles, such as the gastrocnemius and pectoralis major spasticity (12). rESWT delivers energy over a larger, superficial area with a broad treatment range, making it ideal for larger muscle groups or superficial muscles, such as the flexor carpi muscles and biceps brachii spasticity (13, 14). A best practice guideline based on evidence-based medicine supports ESWT with a grade A evidence level, recommending it as an effective intervention for treating PSS (15). Although systematic reviews and meta-analyses have examined the efficacy of ESWT for PSS, the quality of studies varies, outcome measures lack standardization, and existing evidence remains fragmented, complicating clinical decision-making. Furthermore, no studies have specifically addressed these issues. Therefore, this umbrella review systematically evaluates the reporting quality, methodological quality, and evidence quality of relevant systematic reviews and meta-analyses. It further synthesizes evidence on the efficacy and safety of ESWT for PSS, summarizes commonly used ESWT parameter settings and target mechanisms, and provides a reference for developing clinical ESWT treatment protocols for PSS.

## 2 Methods and analysis

The umbrella review adheres to the Preferred Reporting Items for Systematic Reviews and Meta-Analyses (PRISMA) guidelines (16). It has been prospectively registered (PROSPERO: CRD420251124065), and the protocol for this review has been published.

## 3 Requirements for inclusion

We formulated the research question using the PICO framework: among patients with post-stroke spasticity (P), how do rehabilitation outcomes (O) differ between extracorporeal shock wave therapy (I) and conventional rehabilitation or placebo/sham interventions (C)? Eligible control conditions included placebo, sham treatment, and other standard approaches for spasticity management. Outcomes were required to be measured using validated, standardized spasticity assessment instruments. We included only peer-reviewed articles published in English or Chinese and indexed in scientific databases. Studies evaluating combined special interventions were excluded, as were gray literature sources (e.g., unpublished reports), books, conference proceedings, and related materials.

## 4 Search methods, selection of studies, and data extraction

On May 22, 2025, we conducted a comprehensive search of all published literature up to that date. This search covered eight major databases, including PubMed, Web of Science, EMBASE, the Cochrane Database of Systematic Reviews, CNKI, VIP, Sinomed, and Wanfang Data. The specific search strategies for relevant systematic reviews and meta-analyses within each database are detailed in Supplemental Table S1 online.

We systematically reviewed the reference lists of original literature to identify additional relevant studies. Following the AMSTAR 2 (A MeaSurement Tool to Assess systematic Reviews 2) guidelines (17), we employed this tool to assess the methodological quality of included systematic reviews and meta-analyses. The tool comprises 16 assessment items, each corresponding to key criteria evaluating the robustness and validity of systematic reviews. To prevent high scores from masking significant methodological flaws, the AMSTAR 2 development team specifically emphasized the need to focus on seven core domains (items 2, 4, 7, 9, 11, 13, and 15). Each item was rated using a three-level scale: “meets requirements,” “partially meets requirements,” or “does not meet requirements.” Ultimately, the quality of each review was categorized into four levels: high, moderate, low, and very low.

Two independent scholars conducted preliminary screening of identified literature at the title and abstract level, followed by full-text evaluation. In cases of disagreement during screening or evaluation, a third researcher was consulted to reach a final decision. All processes were completed using NoteExpress, a web-based platform specifically designed for literature management. This software incorporates core functional modules for literature collection, classification and organization, reading tracking, and note-taking.

## 5 Data analysis

We systematically extracted and synthesized key characteristics from each review, including study populations, research designs, primary outcome measures, and methodological quality assessments. Subsequently, a narrative synthesis of the findings was conducted. This analysis evaluated intervention effects through indirect comparisons, assessed potential implications for clinical practice, and identified key directions for future research. We verbatim extracted comparative data from the original literature, including effect estimates (e.g., standardized mean differences, mean differences) with corresponding 95% confidence intervals, and measures of heterogeneity (*I*^2^ values reported as percentages).

Given the significant differences in research methodologies and analytical approaches among the selected reviews, conducting new quantitative analyses is no longer feasible. Therefore, we employed a descriptive analysis approach and utilized the literature overlap visualization tool (GROOVE) to visually represent the overlap among the original studies included in the meta-analyses (18). This method assesses the degree of overlap between studies by constructing an evidence matrix and calculating the corrected coverage area (CCA). Statistical techniques categorize the overlap into four levels: slight overlap (CCA < 5%), moderate overlap (6%−10%), high overlap (11%−15%), and very high overlap (≥15%). This systematic approach enables detailed assessment of consistency among different study results, thereby enhancing the interpretive robustness of synthesized evidence.

## 6 Results

### 6.1 Search results

**Figure 1:**
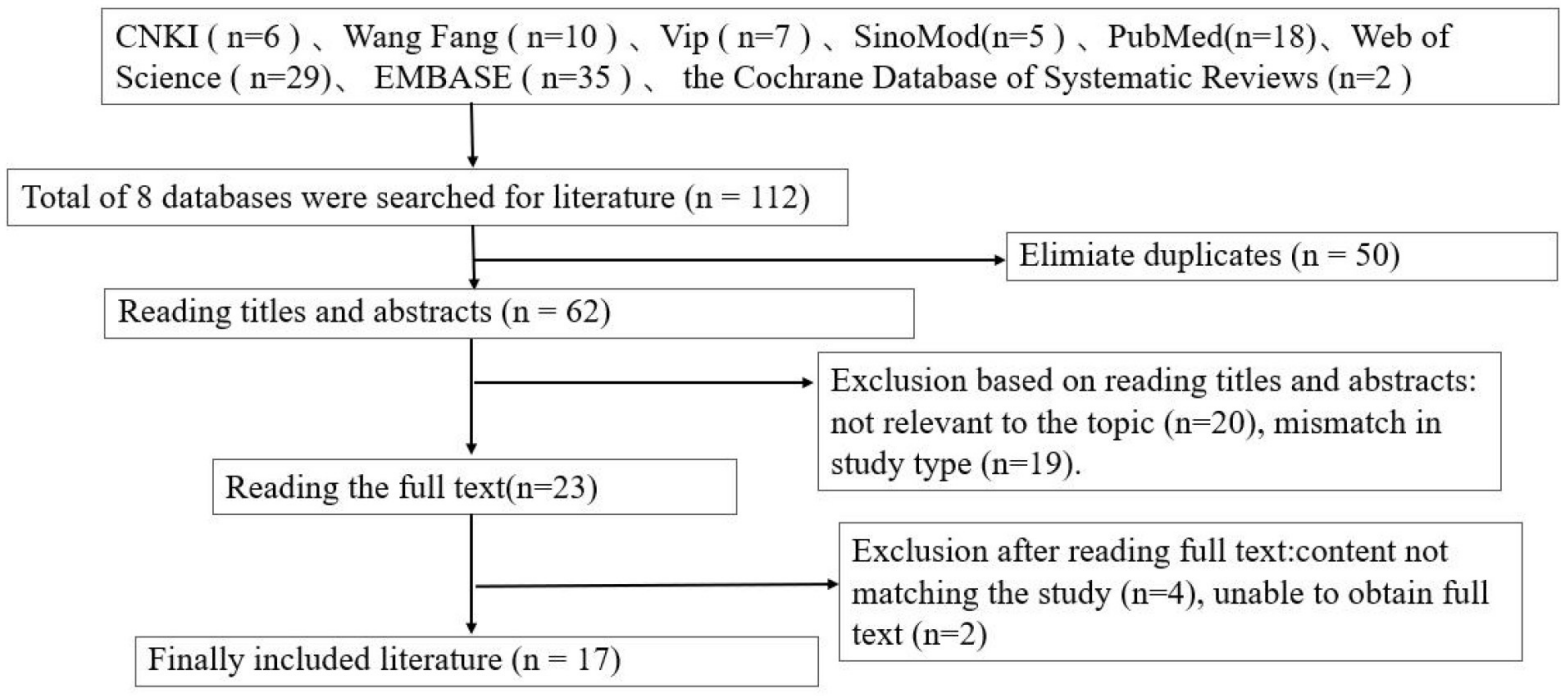
PRISMA flow diagram (preferred reporting items for systematic reviews and meta-analyses).

Through comprehensive literature searches, we initially identified 112 potential studies. Fifty were excluded due to duplication. After preliminary screening of titles and abstracts, an additional 39 studies failed to meet inclusion criteria and were discarded. Subsequent full-text review excluded an additional 6 studies. Following this rigorous screening process, 17 studies were ultimately selected for inclusion: comprising 1 non-randomized controlled trial, 2 quasi-randomized controlled trials, and 14 randomized controlled trials. Figure 1 presents the PRISMA literature screening flowchart, detailing the search and screening steps. Data from all 17 studies meeting inclusion criteria were fully extracted and underwent methodological quality assessment. Excluded studies and their reasons for exclusion are detailed in Supplementary Table S2 online.

### 6.2 Characteristics of the systematic reviews and meta-analyses

The 17 systematic reviews and meta-analyses included in this study were published between 2017 and 2025. Each study evaluated between 6 and 19 research projects, with an average of 11 studies per review. The sample sizes of the included studies ranged from 160 to 871 participants, with an average sample size of 446 participants. Detailed baseline and clinical characteristics of the included studies are presented in Tables 1, 2. The quality of systematic reviews was graded using the AMSTAR-2 assessment tool: 3 high-quality articles (19–21) (17.6%), 2 moderate-quality articles (22, 23) (11.8%), 7 low-quality articles (24–30) (41.2%), and 5 very low-quality articles (31–35) (29.4%). Specific grading criteria are detailed in Supplementary Table S3 online.

**Table 1:** Baseline characteristics of included studies.

Included in the literature	Country	Number of documents/sample size	Age (years)	Average onset	Type	Intervention		Evaluation tool
						Treatment group	Control group	
Guo et al. (24)	China	6/160	44.9–63.15	2.68–53.40 m	nRCTs	ESWT	CRT/none	Newcastle-Ottawa Scale
Guo et al. (25)	China	6/192	48.5–61.7	1.6–66.65 m	RCTs	rESWT ± CRT	CRT ± Sham ESWT	Cochrane Risk of Bias Tool
Xiang et al. (31)	China	8/385	52.28–67.7	/	RCTs	rESWT/ESWT ± CRT	CRT ± Sham ESWT	Cochrane Risk of Bias Tool
Jia et al. (32)	China	8/301	44.11–66.79	3.23–66.65 m	RCTs	ESWT+CRT	CRT ± Sham ESWT	Cochrane Risk of Bias Tool
Liu et al. (33)	China	12/536	/	/	RCTs	rESWT/ESWT ± CRT	CRT ± Sham ESWT	Cochrane Risk of Bias Tool
Mihai et al. (26)	Romania	7/170	44.11–66.9	3.97–99.1 m	RCTs	rESWT/fESWT ± CRT	CRT ± Sham ESWT	PEDro Scale
Cabanas-Valdés et al. (19)	Spain	16/764	46.93–69.72	1.8–100.3 m	RCTs	rESWT/fESWT ± CRT	CRT ± Sham ESWT	PEDro Scale
Cabanas-Valdés et al. (20)	Spain	12/278	25.8–66.9	2.7–99.1 m	RCTs, nRCTs	rESWT/fESWT ± CRT	CRT ± Sham ESWT	PEDro Scale
Ou-Yang et al. (22)	China	13/677	/	Most patients > 6 m	RCTs	rESWT/fESWT	CRT ± Sham ESWT	Cochrane Risk of Bias Tool
Ke et al. (21)	China	11/508	44.11–68.18	2.88–99.1 m	RCTs	rESWT/ESWT ± CRT	CRT ± Sham ESWT	Cochrane Risk of Bias Tool
Li et al. (23)	China	19/871	44.11–68.71	/	RCTs	rESWT/ESWT ± CRT	CRT ± Sham ESWT	Cochrane Risk of Bias Tool
Teng et al. (34)	China	7/257	/	/	RCTs	rESWT ± CRT	CRT ± Sham ESWT	Cochrane Risk of Bias Tool
Chen et al. (35)	China	12/736	50.05–67.25	/	RCTs	ESWT+CRT	CRT	Cochrane Risk of Bias Tool
Afzal et al. (27)	Pakistan	15/389	44.11–66.9	3.43–99.1 m	RCTs, nRCTs	rESWT/fESWT ± CRT	CRT ± Sham ESWT	PEDro Scale
Liu et al. (28)	China	9/327	56–69	Most patients > 6 m	RCTs	rESWT/fESWT+CRT	CRT ± Sham ESWT	Cochrane Risk of Bias Tool
Zhao et al. (29)	China	14/609	49.5–68.18	0.1–105.7 m	RCTs	rESWT ± CRT	CRT ± Sham ESWT	Cochrane Risk of Bias Tool
Sun et al. (30)	China	11/426	/	/	RCTs	ESWT	CRT/Sham ESWT	Cochrane Risk of Bias Tool

CRT, Conventional Rehabilitation Therapy; rESWT, Radial Extracorporeal Shock Wave Therapy; fESWT, Focused Extracorporeal Shock Wave Therapy.

**Table 2:** Clinical and intervention-related characteristics of included studies.

Included in the literature	Physical parameters	Target site	Target muscle	Outcome measure tool	Outcome indicator
Guo et al. (24)	Number of impulses: 1,500–3,000	Applied directly to the spastic muscle tissue	UE: subscapularis, wrist flexors, finger flexors LE: gastrocnemius, soleus	MAS AE	①⑥
Guo et al. (25)	Number of impulses: 1,500–4,000 Pressure: 1.0–3.5 bar Frequency: 5–10 Hz	mUscle belly, Myotendinous junction	UE: finger flexors, wrist flexors, biceps brachii, pronator teres LE: gastrocnemius, soleus	MAS FMA AE	①②⑥
Xiang et al. (31)	Number of impulses: 1,500–4,000 Frequency: 4–12 Hz Density: 0.03–1.95 mJ/mm^2^	Muscle belly, Myotendinous junction	UE: elbow flexor, wrist flexor, finger flexor, shoulder external rotator muscles LE: knee flexor, plantar flexor, ankle dorsiflexion	MAS MTS GA H/M	①④⑧
Jia et al. (32)	Number of impulses: 1,000–4,000 Pressure:1.0–5.0 bar Frequency:4–12 Hz Density: 0.03–1.95 mJ/mm^2^	Muscle belly, Myotendinous junction	UE: forearm flexors, flexor digitorum superficialis, supraspinatus, subscapularis, biceps brachii, wrist flexors LE: gastrocnemius, ankle plantar flexors	MAS FMA VAS GA	①②③④
Liu et al. (33)	Number of impulses: 1,000–3,000 Pressure:1.0–3.5 bar Frequency:4–10 Hz Density:0.03–0.1 mJ/mm^2^	Muscle belly, Myotendinous junction	UE: finger flexors, wrist flexors, Biceps brachii LE: triceps surae	MAS FMA GA AE TUG	①②④⑥⑨
Mihai et al. (26)	Number of impulses: 1,500–2,000 Frequency:4–10 Hz, Density:0.03–0.34 mJ/mm^2^	Muscle belly, Myotendinous junction	LE: gastrocnemius, soleus muscle, ankle plantar flexor, knee flexor	MAS MTS VAS GA AE H/M TUG	①③⑤⑥⑧⑨
Cabanas-Valdés et al. (19)	Number of impulses: 1,000–6,000 Pressure:1.0–4.0 bar Frequency:4–18 Hz Density:0.03–1.95 mJ/mm^2^	Muscle belly, Myotendinous junction	UE: wrist flexors, interossei muscles, flexor digitorum superficialis, biceps brachii, supraspinatus, subscapularis, triceps brachii	MAS FMA-UE VAS AE	①②③⑥
Cabanas-Valdés et al. (20)	Number of impulses: 1,500–2,000 Frequency:2–10 Hz Density:0.03–0.34 mJ/mm^2^	Muscle belly, Myotendinous junction, Plantar fascia	LE: triceps surae	MAS GA LLF	①④⑨
Ou-Yang et al. (22)	/	Muscle belly, Myotendinous junction	/	MAS MTS FMA	①②
Ke et al. (21)	Number of impulses: 1,500–3,000 Pressure: 1.0–2.0 bar Frequency: 4–10 Hz Density: 0.068–0.1 mJ/mm^2^	Muscle belly, Myotendinous junction	LE: triceps surae, semitendinosus	MAS FMA GA	①②④
Li et al. (23)	Number of impulses: 1,500–3,000 Pressure: 1.0–3.0 bar Frequency: 4–10 Hz Density: 0.03–0.6 mJ/mm^2^	Muscle belly, Myotendinous junction	LE: triceps surae, quadriceps femoris, hamstrings, tibialis posterior	MAS FMA CCS GA AE	①②⑤⑥
Teng et al. (34)	/	Lower extremity	/	MAS FMA VAS GA	①②③⑤
Chen et al. (35)	/	Affected extremity	UE: upper limb flexors LE: triceps surae	MAS FMA GA MBI	①②⑤⑦
Afzal et al. (27)	Number of impulses: 1,500–2,000 Pressure: 1.0–2.5 bar Frequency: 2–10 Hz Density: 0.068–0.340 mJ/mm^2^	Muscle belly, Myotendinous junction	LE: gastrocnemius, soleus, semitendinosus	MAS GA BI TUG LLF 10MWT	①④⑦⑨
Liu et al. (28)	Number of impulses: 1,500–3,000 Pressure: 1.0–5.0 bar Frequency: 4–12 Hz Density: 0.03–0.1 mJ/mm^2^	Muscle belly, Myotendinous junction	/	MAS FMA VAS GA	①②③⑤
Zhao et al. (29)	Number of impulses: 1,000–3,000 Pressure: 1.0–3.0 bar Frequency: 6–10 Hz Density: 0.03 mJ/mm^2^	Muscle belly, Myotendinous junction	LE: triceps surae, quadriceps femoris	MAS FMA-LE GA H/M TUG	①②⑤⑧⑨
Sun et al. (30)	/	Muscle belly, Myotendinous junction.	UE: elbow, wrist, hand, shoulder LE: knee, lower leg (gastrocnemius)	MAS	①

MAS, Modified Ashworth Scale; MTS, Modified Tardieu Scale; FMA, Fugl-Meyer Assessment; VAS, Visual Analog Scale; GA, Goniometer Assessment; H/M, H-Reflex/M-Wave Ratio; AE, Adverse Event; CSS, Composite Spasticity Scale; BI, Barthel Index; MBI, Modified Barthel Index; TUG, Timed Up and Go; LLF, Lower Limb Function;10 MWT, 10-Meter Walk Test; UE, Upper Extremity; LE, Lower Extremity.

① Improvement in spasticity; ② Sensorimotor Function; ③ Pain Intensity; ④ Joint Range of Motion; ⑤ Passive Range of Motion; ⑥ Safety Outcomes; ⑦ Activities of Daily Living; ⑧ Neurophysiological Level; ⑨ Gait Function.

**Figure 2:**
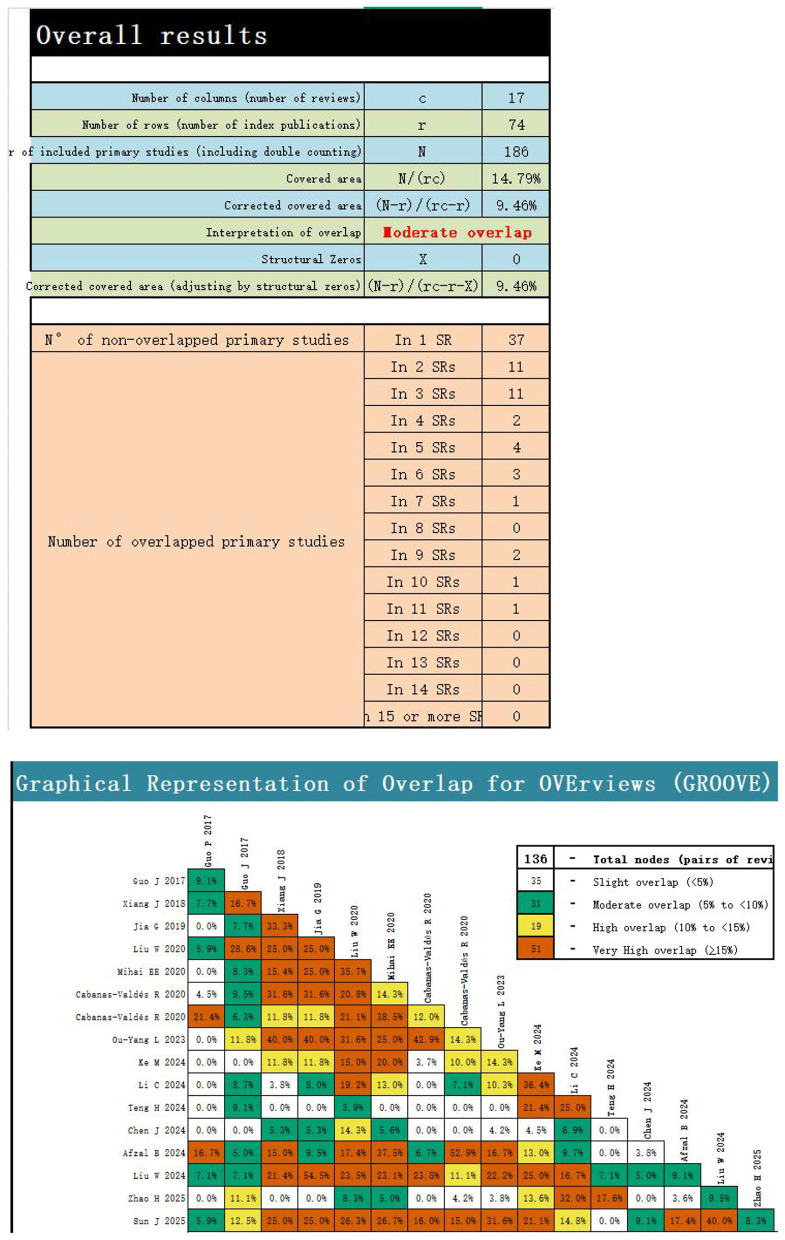
CCA fold diagram (corrected coverage area).

The GROOVE overlap diagram reveals 136 nodes, indicating moderate overlap between systematic reviews and meta-analyses. Among these, 37 original studies appeared in only a single systematic review and meta-analysis, forming the largest group and demonstrating substantial unique evidence. Most studies exhibited low overlap frequency: 32 studies were cited by 2–7 systematic reviews and meta-analyses, while only a small number were frequently cited across multiple reviews. This pattern may be related to the inclusion of a large number of Chinese-language publications. For specific details, refer to Figure 2.

Among the 108 outcomes assessed using the GRADE methodology, 27.8% (30/108) were rated as moderate quality, 36.1% (39/108) as low quality, and 36.1% (39/108) as very low quality. For specific details, see Supplementary Table S4 online.

## 7 Outcomes

Supplementary Table S5 summarizes findings from 17 systematic reviews and meta-analyses, reporting assessed outcomes, pooled effect estimates with 95% confidence intervals, heterogeneity (*I*^2^), and corresponding *p*-values. Across analyses, ESWT was associated with statistically significant improvements in PSS, although the certainty and strength of evidence varied between articles. The included syntheses evaluated a broad range of outcomes, including spasticity severity, sensorimotor function, pain intensity, joint range of motion, passive range of motion and safety outcomes. In addition, we systematically extracted and consolidated intervention characteristics–ESWT modality, treatment parameters, and target sites—to inform evidence-based treatment specifications for PSS management. Notably, most articles highlighted substantial heterogeneity across the original trials and suggested potential publication bias. Owing to differences in outcome measures and pronounced data heterogeneity, a formal meta-analysis was not feasible in the umbrella review. As a result, direct comparisons and quantitative pooling of findings could not be undertaken, despite observed benefits in specific domains such as spasticity and pain relief. Therefore, the evidence was summarized using a narrative synthesis approach.

### 7.1 Improvement in spasticity

Modified Ashworth Scale (MAS). Seventeen studies (19–35) reported on the MAS (evidence quality ranging from moderate to very low), with results indicating ESWT significantly outperformed controls in improving spasticity scores in affected limbs. ESWT significantly reduced MAS scores and muscle tone in PSS patients within less than 12 weeks (19, 20, 22, 24–27, 29–31, 33), demonstrating effective spasticity relief. Cabanas-Valdes et al. (19) observed a gradual decline in effect size over time (from 24 h to over 12 weeks), decreasing from −1.78 to −0.53, suggesting the intervention's immediate effect is strongest and may diminish over time. Notably, Sun et al. (30) demonstrated that long-term improvement (≥4 weeks, MD = −1.15) surpassed short-term improvement (< 4 weeks, MD = −0.75), though substantial heterogeneity may have introduced bias. Most studies indicated ESWT improves spasticity in both upper and lower limbs, with lower limb improvements showing greater consistency. Eleven studies (20, 21, 23, 26–30, 32–34) reported significant improvement in lower limb spasticity, while five studies (19, 25, 28, 30, 32) demonstrated similar effects on upper limb muscle tone, as detailed in Supplementary Table S5.

Modified Tardieu Scale (MTS). Three studies (22, 26, 31) reported on the Modified Tardieu Scale (evidence quality is low), all indicating that ESWT significantly outperformed control groups in improving spasticity scores in affected limbs. Three studies (22, 26, 31) reported significant spasticity improvement within 12 weeks.

### 7.2 Sensorimotor function

Eleven studies (19, 21–23, 25, 28, 29, 32–35) demonstrated that, as measured by the Fugl-Meyer Assessment (FMA), ESWT significantly improved the sensorimotor function of PSS patients compared to the control group (evidence quality ranging from moderate to very low). Four studies (19, 25, 28, 33) evaluated upper limb efficacy, showing significant ESWT effects within less than 12 weeks, particularly pronounced in the early phase (1–2 weeks). However, it is noteworthy that Liu W et al. (33) observed no statistical difference at the 8-week follow-up, suggesting this evidence should be interpreted with caution. Six studies (21, 23, 28, 29, 33, 34) evaluated lower limb efficacy, with five demonstrating significant ESWT effects on lower limb sensory-motor function. While Liu et al. (33) showed no significant effect on the lower limb, this result may be incidental due to the small sample size, and the timeline-related outcomes also require further verification. Additionally, Jia et al. (32), Ou-Yang et al. (22) and Chen et al. (35) did not differentiate between upper and lower limbs. From a temporal perspective, most studies suggest that ESWT improves sensory and motor function more markedly in the short term, with effects potentially diminishing or stabilizing over time. However, this trend may be constrained by sample size limitations and follow-up design, necessitating further evidence to support long-term efficacy.

### 7.3 Pain intensity

Five studies (19, 26, 28, 32, 34) used Visual Analog Scales (VAS) to assess pain intensity (evidence quality ranging from moderate to very low). Notably, three studies (19, 28, 32) demonstrated that ESWT effectively alleviates pain symptoms caused by PSS. This therapy does show efficacy in improving spastic pain after stroke. However, due to the varying quality of the studies, more high-quality randomized controlled trials are needed in the future to validate its long-term efficacy and scope of application.

### 7.4 Joint range of motion and Passive range of motion

Six studies (20, 21, 27, 31–33) evaluated the effect of ESWT on Range Of Motion (ROM) in limbs after stroke (evidence quality ranging from moderate to very low). Five studies (20, 27, 31–33) demonstrated significant efficacy, suggesting ESWT can partially alleviate joint mobility restrictions. Six studies (23, 26, 28, 29, 34, 35) assessed Passive Range Of Motion (PROM) (evidence quality moderate to low), consistently showing PROM improvement. Despite variations in study design and methodological quality, existing evidence generally supports ESWT's role in enhancing ROM/PROM outcomes for PSS management, particularly for patients with significant mobility limitations and impaired active movement capabilities. Future studies with higher quality, more standardized parameter settings, and longer follow-up periods are needed to further validate long-term efficacy and optimize treatment protocols. However, based on current evidence, ESWT represents a promising adjunctive therapeutic option for improving joint mobility and supporting rehabilitation training.

Other outcome measures, such as H/M, TUG, CSS, and MBI, were not subject to descriptive statistical analysis due to limited relevant studies in the literature or insufficient evidence.

## 8 Discussion

This umbrella review has two objectives. First, to rigorously assess the quality of evidence documented in previous systematic reviews and meta-analyses regarding the use of ESWT for treating PSS. Second, to evaluate the consistency and reliability of the translational implications of ESWT for PSS recovery within these systematic reviews and meta-analyses.

### 8.1 Principal findings

Among the 17 included studies, evidence of varying quality indicated that ESWT helps reduce spasticity, improve sensorimotor function, increase active and passive range of motion, and alleviate pain.

Six studies (19, 23–26, 33) reported ESWT-related adverse events during treatment, typically including petechiae, bullae, mild pain, and muscle weakness, though these symptoms usually resolve within a few days. The low incidence of adverse events suggests that the benefits of ESWT outweigh its potential risks for PSS patients. Regarding temporal effects, 17 systematic reviews and meta-analyses utilized the MAS, consistently confirming that ESWT significantly reduces muscle tone in post-stroke spasticity patients within 12 weeks of treatment. Detailed data are presented in Supplementary Table S5 online. This conclusion aligns with the findings of Duan et al. (36).

All studies included in this umbrella review clearly identified the muscle belly-tendon junction as the target site, with mechanisms likely closely related to regulating muscle spindle tension. The selection of target muscles demonstrated distinct clinical specificity: upper limbs primarily focused on the rotator cuff muscles and elbow/wrist flexors, while lower limbs concentrated heavily on the triceps surae, reflecting targeted intervention for common post-stroke spasticity patterns. Details are presented in Table 2. Numerous studies have examined the effects of ESWT on upper and lower limb spasticity, with a particular emphasis on lower limb spasticity. This focus likely stems from the more severe and unique functional impairments associated with lower limb spasticity, coupled with the greater safety and accessibility of ESWT treatment targets in these areas, making the benefit-risk ratio particularly favorable for lower limb applications. Yang et al. (37) demonstrated that ESWT exhibits significant efficacy and safety in alleviating lower limb spasticity (particularly in the ankle plantar flexor muscles) and enhancing limb flexibility. Although studies on upper limb applications have increased in recent years (38, 39), while most show positive outcomes, the overall volume and quality of evidence still fall short of that for lower limb studies. To consolidate the long-term efficacy of ESWT on the limbs, larger-scale studies are urgently needed.

It is noteworthy that existing studies report rather scattered parameter settings for ESWT treatment of PSS. This umbrella review systematically reviewed 17 included publications and summarized commonly used ESWT physical parameters, including number of impulses, pressure, frequency, and density. Results showed that the number of pulses predominantly ranged from 1,500 to 3,000, with 1,500 being the most frequent. Pressure was typically set between 1.0 and 5.0 bar, while frequency primarily ranged from 4 to 10 Hz, with 4 Hz and 5 Hz being the most commonly used. Energy density ranged widely, concentrated between 0.03 and 1.95 mJ/mm^2^. Although ESWT parameters such as energy, frequency, and total shock count should be adjusted based on patient tolerance, disease duration, and tissue type, this compilation provides a foundational reference for future parameter standardization and dose-response studies. Liu et al. (28) indicated that the optimal high-frequency shockwave therapy frequency for treating PSS may be below 8 Hz with pressure < 2 bar. This finding indirectly supports that lower-frequency parameter settings may offer advantages over higher frequencies in improving spasticity. Its potential clinical significance lies in the possibility that low-frequency protocols could enhance tolerability and safety without compromising efficacy. Conversely, excessively high frequencies may induce discomfort or adverse reactions, thereby diminishing overall benefits. Therefore, prioritizing low-to-mid frequency parameters with individualized fine-tuning may represent a more feasible practice approach in PSS rehabilitation settings. Overall, ESWT parameter settings are gradually stabilizing, yet significant variations persist in key parameters such as energy density. Future research should further focus on optimizing parameter mechanisms and validating long-term efficacy sustainability and safety.

### 8.2 Interpretation of study effects

Although the precise mechanisms by which ESWT alleviates PSS remain incompletely defined, a systematic review by Duan et al. (36) suggests that its clinical benefits likely arise from convergent, multi-pathway effects. Available evidence indicates that ESWT may modulate neuromuscular function by inducing nitric oxide (NO) synthesis, reducing acetylcholine release at the neuromuscular junction, and promoting angiogenesis within the muscle–tendon unit (40). In addition, mechanical stimulation of the tendon may attenuate α-motoneuron excitability, as supported by reductions in H-reflex amplitude (41). Collectively, these mechanisms may directly dampen hyperactive spinal stretch reflexes, thereby reducing spasticity and improving joint range of motion (42), providing a key physiological rationale for early intervention. Concurrently, the mechanical loading generated by ESWT can enhance local microcirculation, relieve muscle stiffness and tension, and improve muscle rheological properties (43). This process may facilitate clearance of metabolic by-products while reducing pain, mitigating rigidity, and expanding joint mobility. Taken together, these coordinated effects create a physiological milieu characterized by lower muscle tone, less pain, and greater range of motion–conditions that better support voluntary activity and controlled active motor training, and may ultimately translate into meaningful gains in global sensorimotor function. Overall, the therapeutic effects of ESWT result from the interaction of multiple mechanisms, rather than a single pathway. Through the combined actions of inhibiting nerve conduction, improving local microcirculation, and alleviating muscle stiffness, ESWT improves neuromuscular function, as reflected in clinical outcomes on standardized scales. The reversibility of the neural-related effects explains the short-term nature of improvements in indicators such as MAS, while other effects continue to impact joint mobility and pain control.

A growing body of research evidence suggests that combining ESWT with other interventions may provide more effective relief from PSS and promote functional recovery compared to monotherapy, demonstrating superior comprehensive efficacy. One study (44) demonstrated that combining ESWT with botulinum toxin type A (BoNT-A) reduced MAS by 2 points, rapidly and persistently decreased muscle tone, and sustained efficacy for up to 6 months—compared to only 4 weeks with BoNT-A alone. These findings indicate that the combined ESWT-BoNT-A strategy not only significantly reduces spasticity severity but may also prolong therapeutic duration. Furthermore, studies suggest that initiating ESWT immediately or shortly after BoNT-A injection yields additive benefits in the early phase (45), implying that an “early combined, intensive, or fractionated” approach may enhance short-term efficacy and accelerate functional recovery. Notably, long-term repeated BoNT-A injections may induce neutralizing antibody formation, thereby affecting its biological effects and clinical response. This provides theoretical support for introducing non-pharmacological modalities like ESWT to establish a multimodal treatment framework. In summary, ESWT can serve as an alternative or adjunctive option for PSS treatment within a multimodal comprehensive intervention system; existing evidence generally supports its safety and efficacy in PSS management.

In summary, substantial evidence supports the use of ESWT as an effective and safe adjunctive therapy in the comprehensive management of PSS. Studies indicate that this therapy improves spasticity symptoms, enhances sensorimotor function, alleviates pain, and increases joint flexibility. Existing research provides adequate evidence supporting ESWT for managing PSS, recommending its inclusion as a key component of multimodal rehabilitation. Based on the umbrella review assessed as high or moderate quality using the AMSTAR-2 tool, we propose a recommended pilot protocol: pulse counts of 1,500, frequencies of 4 Hz and 5 Hz, pressure typically set at 1.0–3.0 bar, and energy density concentrated between 0.03 and 0.34 mJ/mm^2^. It is worth noting that optimal ESWT parameters should be tailored to the individual patient, taking into account tolerance, disease duration, and tissue type. High-quality, parameter-stratified randomized controlled trials remain necessary to further optimize intervention strategies.

## 9 Limitations

This umbrella review has several limitations. First, most of the included systematic reviews and meta-analyses had low quality, and the majority of the data were from China, leading to considerable heterogeneity. However, the treatment trends did not substantially differ from those based on English-only literature after incorporating Chinese-language studies. Second, the search strategy was limited to Chinese and English language publications. It remains unclear whether meta-analyses published in other languages would affect our findings. Third, the results may be influenced by overlap among the original studies. Fourth, the included studies rarely mentioned or analyzed outcomes such as H/M, TUG, CSS, and MBI. Lastly, the outcome measures used in the studies were primarily scales, which are inherently subjective. Future research on ESWT for post-stroke spasticity should increasingly adopt objective measurement methods to enhance the quality of evidence. For instance, combining electrophysiological monitoring (such as surface electromyography and H-reflex-related indicators) with biomechanical/robotic assessments (including joint resistance, muscle stiffness, and kinematic metrics) can yield more objective and reproducible efficacy evaluations.

## Data Availability

The original contributions presented in the study are included in the article/Supplementary material, further inquiries can be directed to the corresponding author.
