# Supplementary material for: Extracorporeal shock wave therapy for post-stroke spasticity: an umbrella review of systematic reviews and meta-analyses
Source: Front Neurol. 2026 Apr 20;17:1705104. doi: 10.3389/fneur.2026.1705104 (PMC13135996; doi:10.3389/fneur.2026.1705104)
Supplement: Supplementary file 1 [file Table_1.docx]

Supplementary Material

**Table S1 Search Strategy for the Umbrella Review**

| **PubMed** | #1"stroke"[MeSH]  #2"stroke"[Title/Abstract] OR "apoplexy"[Title/Abstract] OR "cerebrovascular disease"[Title/Abstract] OR "cerebral infarction"[Title/Abstract] OR "cerebral hemorrhage"[Title/Abstract] OR "cerebrovascular attack" [Title/Abstract] OR "cerebrovascular accident"[Title/Abstract]  #3"Spasm"[Mesh]  #4 Muscle Spasticity[Mesh]  #5"spasm"[Title/Abstract] OR "Muscle Spasticity"[Title/Abstract] OR "Muscle Spasm"[Title/Abstract] OR "Spasms,Muscle"[Title/Abstract] OR "Muscular Spasm"[Title/Abstract] OR "Spasm, Muscular"[Title/Abstract] OR "spasticity"[Title/Abstract] OR "Cramp"[Title/Abstract] OR "hypertonia"[Title/Abstract]  #6"Extracorporeal Shockwave Therapy"[MeSH]  #7"Extracorporeal Shockwave Therapy"[Title/Abstract] OR "Shockwave Therapy, Extracorporeal"[Title/Abstract] OR " Extracorporeal Shock Wave Therapy"[Title/Abstract] OR "Shock Wave Therapy"OR "ESWT"[Title/Abstract]  #8"meta-Analysis as Topic"[MeSH Terms]OR "meta-analysis"[Publication Type]OR "meta-analysis"[Title/Abstract] OR "systematic review"[Title/Abstract]  #9 #1OR#2  #10 #3OR #4 OR #5  #11 #6OR #7  #12 #8AND#9AND#10AND#11 |
| --- | --- |
| **EMBASE** | #1'cerebrovascular accident'/exp  #2 'stroke':ab,ti OR 'apoplexy':ab,ti OR 'cerebrovascular disease':ab,ti OR 'cerebral infarction':ab,ti OR 'cerebral hemorrhage':ab,ti OR 'cerebrovascular attack':ab,ti OR 'cerebrovascular accident':ab,ti  #3 #1 OR #2  #4 'spasticity'/exp OR 'spasm'/exp  #5 'muscle spasticity':ab,ti OR 'spastic':ab,ti OR 'spastic disease':ab,ti OR 'spasticism':ab,ti OR 'spasticity':ab,ti OR 'spasm':ab,ti OR 'muscle spasm':ab,ti OR 'spasms,muscle':ab,ti OR 'muscular spasm':ab,ti OR 'spasm, muscular':ab,ti OR 'cramp':ab,ti OR 'hypertonia':ab,ti  #6 #4 OR #5  #7 'shock wave therapy'/exp  #8 'extracorporeal shock wave therapy':ab,ti OR 'extracorporeal shockwave therapy':ab,ti OR 'shock wave treatment':ab,ti OR 'shockwave therapy':ab,ti OR 'shockwave treatment':ab,ti OR 'shock wave therapy':ab,ti  #9 #7 OR #8  #10 'meta analysis'/exp OR 'systematic review'/exp  #11 'meta-analysis':ti,ab OR 'systematic review':ti,ab OR 'meta analy*':ti,ab OR 'metanaly*':ti,ab OR 'metaanaly*':ti,ab OR 'met analy*':ti,ab OR 'systematic review*':ti,ab  #12 #10 OR #11  #13 #3 AND #6 AND #9 AND#12 |
| **Web of Science** | #1 TS=("Stroke" OR "apoplexy" OR "cerebrovascular disease" OR "cerebral infarction" OR "cerebral hemorrhage" OR "cerebrovascular attack" OR "cerebrovascular accident" )  #2 TS=("Extracorporeal Shockwave Therapy" OR "Shockwave Therapy, Extracorporeal" OR " Extracorporeal Shock Wave Therapy" OR "Shock Wave Therapy" OR "ESWT")  #3 TS=('muscle spasticity' OR 'spastic' OR 'spastic disease' OR 'spasticism' OR 'spasticity' OR 'Spasm' OR 'Muscle Spasm' OR 'Spasms,Muscle' OR 'Muscular Spasm' OR 'Spasm, Muscular' OR 'Cramp' OR 'hypertonia' )  TS=(Meta-Analysis OR systematic OR systematic review OR meta-analysis as topic OR metaanaly* OR met analy*) #4 AND #3 AND #2 AND #1 |
| **the Cochrane Database of Systematic Reviews** | #1 MeSH descriptor: [Stroke] explode all trees  #2 ("Stroke" OR "apoplexy" OR "cerebrovascular disease" OR "cerebral infarction" OR "cerebral hemorrhage" OR "cerebrovascular attack" OR "cerebrovascular accident"):ti,ab,kw  #3 #1 OR #2 84649  #4 MeSH descriptor: [Muscle Spasticity] explode all trees  #5 MeSH descriptor: [Spasm] explode all trees  #6 ("Spasm" OR "Muscle Spasm" OR "Spasms,Muscle" OR "Muscular Spasm" OR "Spasm, Muscular" OR "spasticity" OR "Cramp" OR "hypertonia" OR "muscle spasticity" OR "spastic" OR "spastic disease" OR "spasticism" OR "spasticity"):ti,ab,kw  #7 #4 OR #5 OR #6  #8 MeSH descriptor: [Extracorporeal Shockwave Therapy] explode all trees  #9 ("Extracorporeal Shockwave Therapy" OR "Shockwave Therapy, Extracorporeal" OR " Extracorporeal Shock Wave Therapy" OR "Shock Wave Therapy" OR "ESWT"):ti,ab,kw  #10 #8 OR #9 2115  #11 MeSH descriptor: [Meta-Analysis as Topic] explode all trees  #12 (Meta-Analysis OR systematic OR systematic review OR meta-analysis as topic OR metaanaly* OR met analy*):ti,ab,kw  #13 #11 OR #12  #14 #3 AND #7 AND #10 AND #13 |
| **VIP** | [((((((((((题名或关键词=卒中 OR 题名或关键词=脑卒中) OR 题名或关键词=中风) OR 题名或关键词=脑梗死) OR 题名或关键词=脑栓塞) OR 题名或关键词=缺血性脑卒中) OR 题名或关键词=脑出血) OR 题名或关键词=脑血管意外) AND (((题名或关键词=体外冲击波 OR 题名或关键词=冲击波疗法) OR 题名或关键词=冲击波治疗) OR 题名或关键词=体外冲击波疗法)) AND (((((题名或关键词=痉挛 OR 题名或关键词=肌肉痉挛) OR 题名或关键词=肌痉挛) OR 题名或关键词=痉挛发作) OR 题名或关键词=肢体痉挛) OR 题名或关键词=痉挛性瘫痪)) AND (((题名或关键词=系统评价 OR 题名或关键词=系统综述) OR 题名或关键词=荟萃分析) OR 题名或关键词=Meta分析))](https://qikan.cqvip.com/Qikan/search/index?LngMySearHistoryIdGuid=348736cd-9e41-42b3-aa99-1c6f16cfacfa&from=Qikan_Article_History) |
| **CNKI** | 卒中 + 脑卒中 + 中风 + 脑梗死 + 脑栓塞 + 缺血性脑卒中 + 脑出血 + 脑血管意外  体外冲击波 + 冲击波疗法 + 冲击波治疗 + 体外冲击波疗法  痉挛 + 肌肉痉挛 + 肌痉挛 + 痉挛发作 + 肢体痉挛 + 痉挛性瘫痪  系统评价 + 系统综述 + 荟萃分析 + Meta分析 |
| **WangFANG** | 卒中OR 脑卒中OR中风 OR 脑梗死 OR 脑栓塞 OR 缺血性脑卒中 OR 脑出血 OR 脑血管意外  体外冲击波 OR 冲击波疗法OR冲击波治疗 OR体外冲击波疗法  痉挛 OR 肌肉痉挛 OR 肌痉挛 OR痉挛发作 OR 肢体痉挛 OR痉挛性瘫痪  系统评价OR系统综述 OR 荟萃分析 OR Meta分析 |
| **Sinomed** | ( "卒中"[常用字段:智能] OR "脑卒中"[常用字段:智能] OR "中风"[常用字段:智能] OR "脑梗死"[常用字段:智能] OR "脑栓塞"[常用字段:智能] OR "缺血性脑卒中"[常用字段:智能] OR "脑出血"[常用字段:智能] OR "脑血管意外"[常用字段:智能]) AND( "体外冲击波"[常用字段:智能] OR "冲击波疗法"[常用字段:智能] OR "冲击波治疗"[常用字段:智能] OR "体外冲击波疗法"[常用字段:智能]) AND( "痉挛"[常用字段:智能] OR "肌肉痉挛"[常用字段:智能] OR "肌痉挛"[常用字段:智能] OR "痉挛发作"[常用字段:智能] OR "肢体痉挛"[常用字段:智能] OR "痉挛性瘫痪"[常用字段:智能]) AND( "系统评价"[常用字段:智能] OR "系统综述"[常用字段:智能] OR "荟萃分析"[常用字段:智能] OR "Meta分析"[常用字段:智能]) |

|  |
| --- |

**
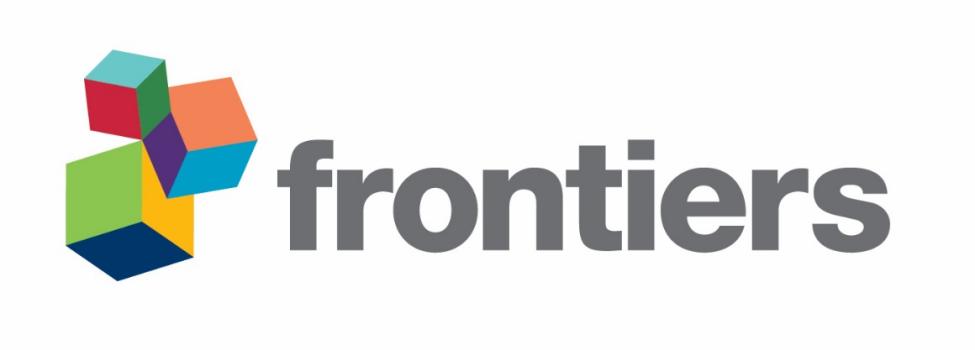
**
