# Supplementary material for: Extracorporeal shock wave therapy for post-stroke spasticity: an umbrella review of systematic reviews and meta-analyses
Source: Front Neurol. 2026 Apr 20;17:1705104. doi: 10.3389/fneur.2026.1705104 (PMC13135996; doi:10.3389/fneur.2026.1705104)
Supplement: Supplementary file 2 [file Table_2.docx]

Supplementary Material

**Table S2 Research articles not included in the analysis, along with the reasoning behind their exclusion.**

| **References** | **Reasons for exclusion** |
| --- | --- |
| Hsu, P. C., Chang, K. V., Chiu, Y. H., Wu, W. T., & Özçakar, L. (2021). Comparative Effectiveness of Botulinum Toxin Injections and Extracorporeal Shockwave Therapy for Post-Stroke Spasticity: A Systematic Review and Network Meta-Analysis. EClinicalMedicine, 43, 101222. https://doi.org/10.1016/j.eclinm.2021.101222 | Not a systematic review |
| Zhang, T., & Zhang, C. (2023). Extracorporeal shock wave therapy for shoulder pain after stroke: A systematic review and meta-analysis. Clinical rehabilitation, 37(6), 774–790. https://doi.org/10.1177/02692155231152134 | Wrong population |
| Du, Y. N., Li, Y., Zhang, T. Y., Jiang, N., Wei, Y., Cheng, S. H., Li, H., & Duan, H. Y. (2024). Efficacy of botulinum toxin A combined with extracorporeal shockwave therapy in post-stroke spasticity: a systematic review. Frontiers in neurology, 15, 1342545.  https://doi.org/10.3389/fneur.2024.1342545 | Wrong study design |
| de Roo, E. G., Koopman, S. B., Janssen, T. W., & Aertssen, W. F. M. (2025). The effects of extracorporeal shock wave therapy in children with cerebral palsy: a systematic review. International journal of surgery (London, England), 111(4), 2773–2790. https://doi.org/10.1097/JS9.0000000000002251 | Wrong population |
| Zhang, X., & Ma, Y. (2023). Global trends in research on extracorporeal shock wave therapy (ESWT) from 2000 to 2021. BMC musculoskeletal disorders, 24(1), 312.  https://doi.org/10.1186/s12891-023-06407-9 | Not a systematic review |
| Chang, M. C., Choo, Y. J., Kwak, S. G., Nam, K., Kim, S. Y., Lee, H. J., & Kwak, S. (2023). Effectiveness of Extracorporeal Shockwave Therapy on Controlling Spasticity in Cerebral Palsy Patients: A Meta-Analysis of Timing of Outcome Measurement. Children (Basel, Switzerland), 10(2), 332. https://doi.org/10.3390/children10020332 | Wrong population |
| Cidral-Filho, F., Donatello, N. N., Lugtu, C., & Hewitson, A. (2024). Photobiomodulation on shoulder and neck pain and disability: A comprehensive review. Lasers in medical science, 39(1), 263.  https://doi.org/10.1007/s10103-024-04212-x | Not a systematic review |
| Bian, M., Chen, F., Su, H., Li, Z., Sun, X., Liu, Y., Shi, J., Liu, S., & Rong, R. (2025). Comparison of the effects of different physical stimulation therapies on reducing upper limb spastic paralysis and motor dysfunction in stroke survivors after stroke: a network meta-analysis of randomized controlled trials. Frontiers in neurology, 16, 1554583.  https://doi.org/10.3389/fneur.2025.1554583 | Not a systematic review |
| Otero-Luis, I., Cavero-Redondo, I., Álvarez-Bueno, C., Martinez-Rodrigo, A., Pascual-Morena, C., Moreno-Herráiz, N., & Saz-Lara, A. (2024). Effectiveness of Extracorporeal Shock Wave Therapy in Treatment of Spasticity of Different Aetiologies: A Systematic Review and Meta-Analysis. Journal of clinical medicine, 13(5), 1323.  https://doi.org/10.3390/jcm13051323 | Wrong population |
| Chen, B., Yang, T., Liao, Z., Sun, F., Mei, Z., & Zhang, W. (2025). Pathophysiology and Management Strategies for Post-Stroke Spasticity: An Update Review. International journal of molecular sciences, 26(1), 406. https://doi.org/10.3390/ijms26010406 | Not a systematic review |

|  |
| --- |

**
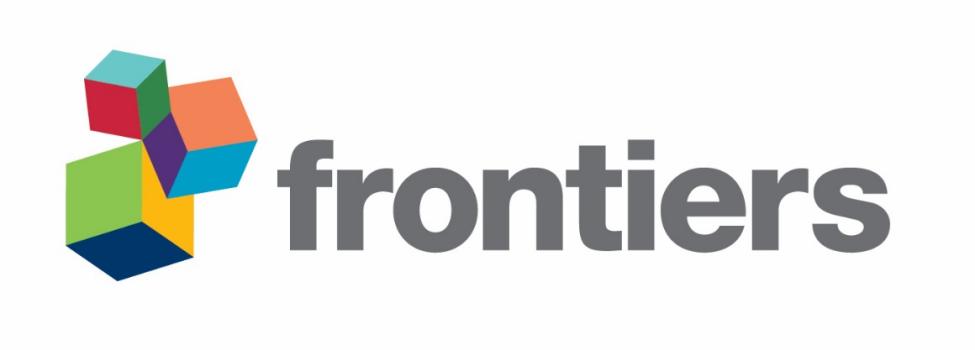
**
