# Supplementary material for: Extracorporeal shock wave therapy for post-stroke spasticity: an umbrella review of systematic reviews and meta-analyses
Source: Front Neurol. 2026 Apr 20;17:1705104. doi: 10.3389/fneur.2026.1705104 (PMC13135996; doi:10.3389/fneur.2026.1705104)
Supplement: Supplementary file 3 [file Table_3.docx]

Supplementary Material

**Table S3.AMSTAR2 Methodological Quality Assessment Results**

| Included in the literature | Q1 | Q2* | Q3 | Q4* | Q5 | Q6 | Q7* | Q8 | Q9* | Q10 | Q11* | Q12 | Q13* | Q14 | Q15* | Q16 | quality assessment |
| --- | --- | --- | --- | --- | --- | --- | --- | --- | --- | --- | --- | --- | --- | --- | --- | --- | --- |
| Guo P et al.2017 | Y | N | PY | Y | Y | Y | Y | Y | PY | N | Y | N | Y | Y | Y | Y | Low |
| Guo J et al.2017 | Y | N | PY | Y | Y | Y | Y | Y | PY | N | Y | N | Y | Y | Y | Y | Low |
| Xiang J et al.2018 | Y | N | N | PY | Y | Y | PY | Y | Y | N | Y | N | PY | Y | N | Y | Very Low |
| Jia G et al.2019 | Y | N | PY | Y | Y | Y | Y | Y | Y | N | Y | PY | PY | Y | N | Y | Very Low |
| Liu W et al.2020 | Y | N | Y | PY | Y | Y | Y | Y | Y | N | Y | PY | PY | Y | N | Y | Very Low |
| Mihai EE et al. 2020 | Y | Y | Y | Y | Y | Y | PY | Y | Y | N | Y | N | Y | Y | N | Y | Low |
| Cabanas-Valdés R et al.2020 | Y | Y | Y | Y | Y | Y | Y | Y | Y | N | Y | PY | Y | Y | Y | Y | High |
| Cabanas-Valdés R et al.2020 | Y | Y | Y | Y | Y | Y | PY | Y | Y | N | Y | PY | Y | Y | Y | Y | High |
| Ou-Yang L et al.2023 | Y | Y | PY | Y | Y | Y | PY | Y | Y | N | Y | N | Y | Y | Y | Y | Moderate |
| Ke M et al.2024 | Y | Y | Y | Y | Y | Y | PY | Y | Y | N | Y | PY | Y | Y | Y | Y | High |
| Li C et al.2024 | Y | Y | Y | Y | Y | Y | PY | Y | Y | N | Y | PY | Y | Y | Y | N | Moderate |
| Teng H et al.2024 | Y | N | N | Y | Y | Y | PY | PY | Y | N | Y | Y | PY | Y | N | Y | Very Low |
| Chen J et al.2024 | Y | N | N | Y | Y | Y | PY | Y | Y | N | Y | PY | PY | N | N | N | Very Low |
| Afzal B et al.2024 | Y | Y | PY | Y | Y | Y | PY | Y | Y | N | Y | N | PY | Y | N | Y | Low |
| Liu W et al.2024 | Y | N | Y | Y | Y | Y | PY | Y | Y | N | Y | N | Y | Y | Y | Y | Low |
| Zhao H et al.2025 | Y | N | Y | Y | Y | Y | PY | Y | Y | N | Y | N | PY | Y | Y | N | Low |
| Sun J et al.2025 | Y | N | Y | PY | Y | Y | PY | Y | Y | N | Y | Y | PY | Y | Y | PY | Low |

Note: Y: Yes; N: No; PY: Partially yes. * denotes key items. Q1: Includes all PICO elements; Q2: Registered study protocol; Q3: Explains rationale for study types included; Q4: Comprehensive literature search; Q5: Two-person independent literature screening; Q6: Two-person independent data extraction; Q7: Provides list and rationale for excluded studies; Q8: Detailed description of included studies' key characteristics; Q9: Uses appropriate tools to assess risk of bias; Q10: Reports funding sources for included studies; Q11: Uses appropriate methods for combining results; Q12: Considers potential impact of bias risk; Q13: Discusses bias risk's effect on study results; Q14: Explains heterogeneity within studies; Q15: Investigates publication bias's impact on results; Q16: Reports conflicts of interest and funding details.

|  |
| --- |

**
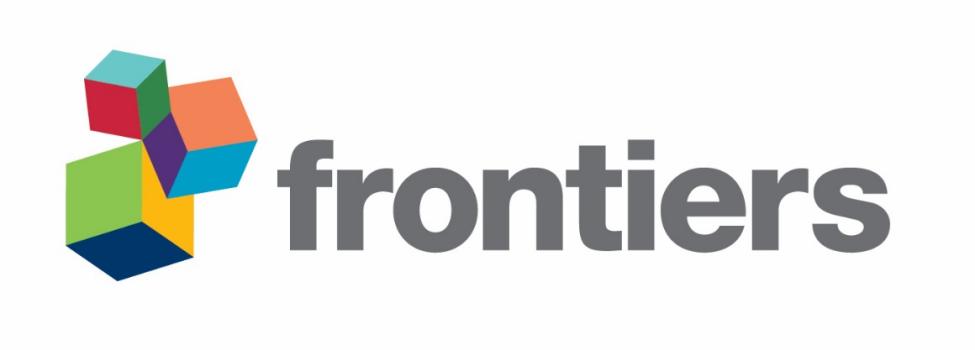
**
