# Supplementary material for: Extracorporeal shock wave therapy for post-stroke spasticity: an umbrella review of systematic reviews and meta-analyses
Source: Front Neurol. 2026 Apr 20;17:1705104. doi: 10.3389/fneur.2026.1705104 (PMC13135996; doi:10.3389/fneur.2026.1705104)
Supplement: Supplementary file 4 [file Table_4.docx]

Supplementary Material

**Table S4. Results of the assessment of the quality of evidence for each outcome of the included meta-analyses using GRADE**

| Studies ID  (Author) | Outcomes | supplement | GRADE items | | | | | Quality of the evidence |
| --- | --- | --- | --- | --- | --- | --- | --- | --- |
|  |  |  | Risk of bias | Inconsistency | Indirectness | Imprecision | Publication bias |  |
| E1 Guo P et al.2017 | MAS | Immediately | Very serious (-2) | Very serious (-2) | Neutral | Neutral | Neutral | Very Low |
|  |  | Long-term (4 w) | Very serious (-2) | Very serious (-2) | Neutral | Neutral | Neutral | Very Low |
| E2 Guo J et al.2017 | Upper limb MAS | Immediate | Serious(-1) | Very serious (-2) | Neutral | Neutral | Neutral | Very Low |
|  |  | Short-term (≤3 m) | Serious(-1) | Very serious (-2) | Neutral | Neutral | Neutral | Very Low |
|  | Upper limb FMA | Immediate | Very serious (-2) | Neutral | Neutral | Neutral | Neutral | Low |
|  |  | Short-term (≤3 m) | Very serious (-2) | Very serious (-2) | Neutral | Very serious (-2) | Neutral | Very Low |
|  | Lower limb MAS | Immediate | Very serious (-2) | Serious (-1) | Neutral | Serious (-1) | Neutral | Very Low |
| E3 Xiang J et al.2018 | MAS | \ | Serious(-1) | Very serious (-2) | Neutral | Neutral | Neutral | Very Low |
|  |  | Long-term (4 w) | Serious(-1) | Very serious (-2) | Neutral | Serious (-1) | Neutral | Very Low |
|  | MTS | Immediate | Serious(-1) | Serious(-1) | Neutral | Neutral | Neutral | Low |
|  | H/M | Immediate | Serious(-1) | Very serious (-2) | Neutral | Neutral | Neutral | Very Low |
|  | ROM | Immediate | Serious(-1) | Very serious (-2) | Neutral | Neutral | Neutral | Very Low |
| E4 Jia G et al.2019 | MAS | Upper limb | Serious(-1) | Very serious (-2) | Neutral | Neutral | Neutral | Very Low |
|  |  | Lower limb | Serious(-1) | Neutral | Neutral | Neutral | Neutral | Moderate |
|  | VAS | \ | Serious(-1) | Neutral | Neutral | Neutral | Neutral | Moderate |
|  | ROM | \ | Serious(-1) | Neutral | Neutral | Neutral | Neutral | Moderate |
|  | FMA | \ | Serious(-1) | Very serious (-2) | Neutral | Neutral | Neutral | Very Low |
| E5 Liu W et al.2020 | Lower limb MAS | Immediate | Serious(-1) | Neutral | Neutral | Serious(-1) | Neutral | Low |
|  |  | 1w | Serious(-1) | Neutral | Neutral | Serious(-1) | Neutral | Low |
|  |  | 3-4w | Serious(-1) | Neutral | Neutral | Neutral | Neutral | Moderate |
|  |  | 12w | Serious(-1) | Very serious (-2) | Neutral | Neutral | Neutral | Very Low |
|  | Upper limb FMA | 1-2w | Serious(-1) | Serious(-1) | Neutral | Neutral | Neutral | Low |
|  |  | 3-4w | Serious(-1) | Neutral | Neutral | Neutral | Neutral | Moderate |
|  |  | 8w | Serious(-1) | Very serious (-2) | Neutral | Serious(-1) | Neutral | Very Low |
|  | Lower limb FMA | 1w | Serious(-1) | Very serious (-2) | Neutral | Serious(-1) | Neutral | Very Low |
|  |  | 4w | Serious(-1) | Very serious (-2) | Neutral | Serious(-1) | Neutral | Very Low |
|  | Lower limb ROM | Immediate | Serious(-1) | Neutral | Neutral | Serious(-1) | Neutral | Low |
|  |  | 1w | Serious (-1) | serious (-1) | Neutral | Serious(-1) | Serious(-1) | Very Low |
|  |  | 3-4w | Serious(-1) | Neutral | Neutral | Neutral | Serious(-1) | Low |
|  |  | 12w | Serious(-1) | Very serious (-2) | Neutral | Neutral | Serious(-1) | Very Low |
|  | TUG | Immediate | Serious(-1) | Very serious (-2) | Neutral | Neutral | Serious(-1) | Very Low |
|  |  | 1w | Serious(-1) | Neutral | Neutral | Neutral | Serious(-1) | Low |
|  |  | 3-4w | Serious(-1) | Neutral | Neutral | Serious(-1) | Serious(-1) | Very Low |
|  |  | 12w | Serious(-1) | Very serious (-2) | Neutral | Serious(-1) | Serious(-1) | Very Low |
| E6 Mihai EE et al. 2020 | MAS | Short-term (1 w) | Serious(-1) | Neutral | Neutral | Neutral | Neutral | Moderate |
|  |  | Long-term (3-12 w) | Serious(-1) | Serious(-1) | Neutral | Neutral | Neutral | Low |
|  |  | between-group comparison. (Long-term) | Serious(-1) | Neutral | Neutral | Serious(-1) | Neutral | Low |
|  | MTS | Long-term (3-12 w) | Neutral | Neutral | Serious(-1) | Serious(-1) | Neutral | Low |
|  | H/M | \ | Serious(-1) | Neutral | Neutral | Serious(-1) | Neutral | Low |
|  | VAS | Long-term (3-12 w): | Serious(-1) | Neutral | Neutral | Serious(-1) | Neutral | Low |
|  | PROM | Long-term (3-12 w) | Serious(-1) | Neutral | Neutral | Neutral | Neutral | Moderate |
|  | TUG | \ | Serious(-1) | Neutral | Neutral | Serious(-1) | Neutral | Low |
| E7 Cabanas-Valdés R et al.2020 | MAS | <24h | Neutral | Very serious (-2) | Neutral | Neutral | Neutral | Low |
|  |  | 24h-3w | Neutral | Very serious (-2) | Neutral | Neutral | Neutral | Low |
|  |  | 4-12w | Neutral | Very serious (-2) | Neutral | Neutral | Neutral | Low |
|  |  | >12w | Neutral | Very serious (-2) | Neutral | Neutral | Neutral | Low |
|  | Upper limb FMA | 24h-3w | Neutral | Very serious (-2) | Neutral | Neutral | Neutral | Low |
|  |  | 4-12w | Neutral | Very serious (-2) | Neutral | Neutral | Neutral | Low |
|  |  | >12w | Neutral | Very serious (-2) | Neutral | Neutral | Neutral | Low |
|  | VAS | 24h | Neutral | Very serious (-2) | Neutral | Neutral | Neutral | Low |
|  |  | 24h-3w | Neutral | serious (-1) | Neutral | Neutral | Neutral | Moderate |

| E8 Cabanas-Valdés R et al.2020 | Lower Limb MAS | Short-term | Serious(-1) | Neutral | Neutral | Neutral | Neutral | Moderate |
| --- | --- | --- | --- | --- | --- | --- | --- | --- |
|  |  | mid-term | Serious (-1) | Very serious (-2) | Neutral | Neutral | Neutral | Very Low |
|  |  | Long-term | Serious(-1) | Neutral | Neutral | Neutral | Neutral | Moderate |
|  | ROM | Short-term | Serious (-1) | Neutral | Neutral | Neutral | Neutral | Moderate |
|  |  | mid-term | Serious (-1) | Very serious (-2) | Neutral | Serious(-1) | Neutral | Very Low |
|  |  | Long-term | Serious(-1) | Neutral | Neutral | Serious(-1) | Neutral | Low |
|  | Lower Limb Function | mid-term | Serious(-1) | Neutral | Neutral | Serious(-1) | Serious(-1) | Very Low |
|  |  | Long-term | Serious(-1) | Neutral | Neutral | Serious(-1) | Serious(-1) | Very Low |
| E9 Ou-Yang L et al.2023 | MAS | Short-term(<2w) | Neutral | Very serious (-2) | Neutral | Neutral | Neutral | Low |
|  |  | mid-term (2w-4w) | Neutral | Very serious (-2) | Neutral | Neutral | Neutral | Low |
|  |  | Long-term (4w and ≤3m) | Neutral | Very serious (-2) | Neutral | Neutral | Neutral | Low |
|  | MTS | Short-term(<2w) | Serious(-1) | Neutral | Neutral | Serious(-1) | Neutral | Low |
|  |  | mid-term (2w and ≤4w) | Serious(-1) | Neutral | Neutral | Neutral | Neutral | Low |
|  | FMA | Short-term(<2w) | Neutral | Very serious (-2) | Neutral | Neutral | Neutral | Low |
|  |  | mid-term (2w and ≤4w) | Neutral | Very serious (-2) | Neutral | Serious(-1) | Neutral | Very Low |
|  |  | Long-term (4w and ≤3m) | Neutral | Very serious (-2) | Neutral | Serious(-1) | Neutral | Very Low |
| E10 Ke M et al.2024 | MAS | \ | Serious(-1) | Neutral | Neutral | Neutral | Neutral | Moderate |
|  | FMA | \ | Serious(-1) | Neutral | Neutral | Neutral | Neutral | Moderate |
|  | ROM | \ | Serious(-1) | Neutral | Neutral | Neutral | Neutral | Moderate |
| E11 Li C et al.2024 | MAS | \ | Serious(-1) | Neutral | Neutral | Neutral | Neutral | Moderate |
|  | FMA-LE | \ | Serious(-1) | Very serious (-2) | Neutral | Neutral | Neutral | Very Low |
|  | CSS | \ | Serious(-1) | Very serious (-2) | Neutral | Neutral | Neutral | Very Low |
|  | PROM | \ | Serious(-1) | Neutral | Neutral | Neutral | Neutral | Moderate |
| E12 Teng H et al.2024 | MAS | \ | Serious(-1) | Neutral | Neutral | Neutral | Neutral | Moderate |
|  | FMA-LE | \ | Serious(-1) | Neutral | Neutral | Neutral | Neutral | Moderate |
|  | PROM | \ | Serious(-1) | Neutral | Neutral | Neutral | Neutral | Moderate |
|  | VAS | \ | Serious(-1) | Very serious (-2) | Neutral | Serious(-1) | Neutral | Very Low |
| E13 Chen J et al.2024 | MAS | \ | Serious(-1) | Very serious (-2) | Neutral | Neutral | Neutral | Very Low |
|  | FMA | \ | Serious(-1) | Very serious (-2) | Neutral | Neutral | Neutral | Very Low |
|  | MBI | \ | Serious(-1) | Very serious (-2) | Neutral | Neutral | Neutral | Very Low |
|  | PROM | \ | Serious(-1) | Very serious (-2) | Neutral | Neutral | Neutral | Very Low |
| E14 Afzal B et al.2024 | MAS | Short-term | Serious (-1) | Neutral | Neutral | Serious(-1) | Neutral | Low |
|  |  | Long-term | Serious (-1) | Neutral | Neutral | Neutral | Neutral | Moderate |
|  | ROM | Short-term | Serious (-1) | Neutral | Neutral | Serious(-1) | Neutral | Low |
|  |  | Long-term | Serious (-1) | Neutral | Neutral | Neutral | Neutral | Moderate |
|  | Lower limb function | Short-term | Serious(-1) | Neutral | Neutral | Neutral | Neutral | Moderate |
|  |  | Long-term | Serious (-1) | Neutral | Neutral | Neutral | Neutral | Moderate |
|  | TUG | \ | Serious(-1) | Neutral | Neutral | Very serious (-2) | Neutral | Very Low |
|  | 10MWT | Long-term | Serious(-1) | Very serious (-2) | Neutral | Serious(-1) | Serious(-1) | Very Low |
|  | BI | Long-term | Serious (-1) | Neutral | Neutral | Neutral | Serious(-1) | Low |
| E15 Liu W et al.2024 | MAS | Upper limb | Serious(-1) | Serious(-1) | Neutral | Neutral | Neutral | Low |
|  |  | Lower limb | Serious(-1) | Neutral | Neutral | Neutral | Neutral | Moderate |
|  | VAS | Upper limb | Serious(-1) | Serious(-1) | Neutral | Neutral | Neutral | Low |
|  | PROM | Upper limb | Serious(-1) | Very serious (-2) | Neutral | Very serious (-2) | Neutral | Very Low |
|  |  | Lower limb | Serious(-1) | Neutral | Neutral | Neutral | Neutral | Moderate |
|  | FMA | Upper limb | Serious(-1) | Neutral | Neutral | Neutral | Neutral | Moderate |
|  |  | Lower limb | Serious(-1) | Neutral | Neutral | Neutral | Neutral | Moderate |
| E16 Zhao H et al.²2025 | Lower limb MAS (post-treatment) | \ | Serious(-1) | Serious(-1) | Neutral | Neutral | Neutral | Low |
|  | Lower limb MAS (follow-up) | \ | Serious(-1) | Neutral | Neutral | Neutral | Neutral | Moderate |
|  | Lower limb FMA | \ | Serious(-1) | Very serious (-2) | Neutral | Neutral | Neutral | Very Low |
|  | PROM | \ | Serious(-1) | Serious(-1) | Neutral | Neutral | Neutral | Low |
|  | TUG | \ | Serious(-1) | Serious(-1) | Neutral | Neutral | Neutral | Low |
|  | H/M | \ | Serious(-1) | Very serious (-2) | Neutral | serious (-1) | Neutral | Very Low |
| E17 Sun J et al.2025 | MAS | Short-term | Serious (-1) | Serious(-1) | Neutral | Neutral | Neutral | Low |
|  |  | Long-term | Serious (-1) | Neutral | Neutral | Neutral | Neutral | Moderate |
|  |  | Upper limb | Serious(-1) | Serious(-1) | Neutral | Neutral | Neutral | Low |
|  |  | Lower limb | Serious(-1) | Neutral | Neutral | Neutral | Neutral | Moderate |

|  |
| --- |

**
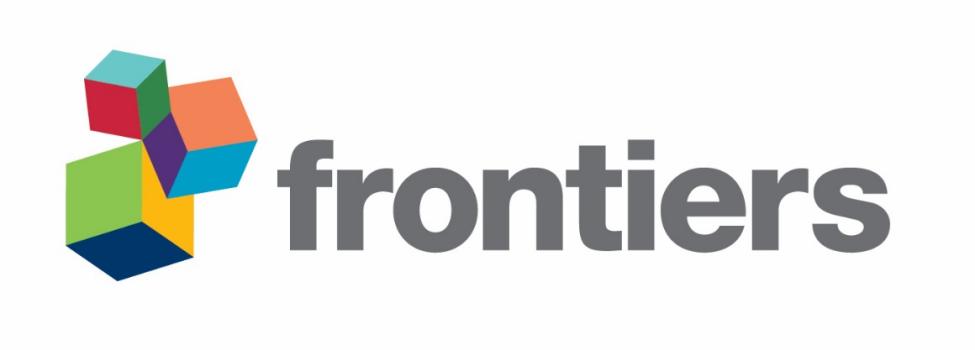
**
