# Supplementary material for: Extracorporeal shock wave therapy for post-stroke spasticity: an umbrella review of systematic reviews and meta-analyses
Source: Front Neurol. 2026 Apr 20;17:1705104. doi: 10.3389/fneur.2026.1705104 (PMC13135996; doi:10.3389/fneur.2026.1705104)
Supplement: Supplementary file 5 [file Table_5.docx]

Supplementary Material

**Table S5. Outcomes of included meta-analyses.**

| **StudiesID (Author)** | **Outcomes** | **Supplement** | **SMDorMDorWMD(95%CI)** | **Effectsize** | **Pvalue** | **I^2^** | **GRADE** |
| --- | --- | --- | --- | --- | --- | --- | --- |
| E1 Guo P et al.2017 | MAS | Immediately | SMD=-1.57(-2.20,-0.94) | Large | <0.001 | 83.8% | Very Low |
|  |  | 4w | SMD=-1.93(-2.71,-1.15) | Large | <0.001 | 83.6% | Very Low |
| E2 Guo J et al.2017 | Upper limb MAS | Immediate | MD=-0.91(-1.58,-0.23) | \ | 0.009 | 97.0% | Very Low |
|  |  | Short-term (≤3 m) | MD=-1.03(-1.27,-0.78) | \ | <0.00001 | 94.0% | Very Low |
|  | Upper limb FMA | Immediate | MD=4.14(0.31,7.96) | \ | 0.03 | 0.0% | Low |
|  |  | Short-term (≤3 m) | MD=5.35(-7.73,18.43) | \ | 0.42 | 78.0% | Very Low |
|  | Lower limb MAS | Immediate | MD=0.10(-1.25,1.45) | \ | 0.88 | 58.0% | Very Low |
| E3Xiang J et al.2018 | MAS | \ | SMD=-1.22(-1.77,-0.66) | Large | <0.001 | 86.7% | Very Low |
|  |  | 4w | SMD=-1.73(-3.99,0.54) | Large | 0.135 | 95.2% | Very Low |
|  | MTS | \ | SMD=0.70(0.42,0.99) | Moderate | <0.001 | 47.2% | Low |
|  | H/M | \ | WMD=-0.76(-1.19,-0.33) | \ | <0.001 | \ | Very Low |
|  | ROM | \ | SMD=0.69(0.06,1.32) | Moderate | 0.031 | 80.6% | Very Low |
| E4 Jia G et al.2019 | MAS | Upper limb | WMD=-0.32(-0.50,-0.13) | \ | 0.0009 | 73.0% | Very Low |
|  |  | Lower limb | WMD=-0.69(-1.08,-0.30) | \ | 0.0006 | 0.0% | Moderate |
|  | VAS | \ | WMD=-0.94(-1.51,-0.37) | \ | 0.001 | 15.0% | Moderate |
|  | ROM | \ | WMD=5.91(2.76,9.18) | \ | <0.001 | 0.0% | Moderate |
|  | FMA | \ | WMD=1.26(0.29,2.24) | \ | 0.01 | 96.0% | Very Low |
| E5 Liu W et al.2020 | Lower limb MAS | Immediate | WMD=-0.28(-0.65,0.10) | \ | 0.15 | 0.0% | Low |
|  |  | 1w | WMD=-0.11(-0.53,0.31) | \ | 0.61 | 0.0% | Low |
|  |  | 3-4w | WMD=-0.45(-0.74,-0.16) | \ | 0.002 | 0.0% | Moderate |
|  |  | 12w | WMD=-0.60(-1.08,-0.12) | \ | 0.01 | \ | Very Low |
|  | Upper limb FMA | 1-2w | WMD=5.22(2.55,7.89) | \ | 0.0001 | 54.0% | Low |
|  |  | 3-4w | WMD=3.75(0.37,7.12) | \ | 0.03 | 0.0% | Moderate |
|  |  | 8w | WMD=2.50(-4.69,9.69) | \ | 0.50 | \ | Very Low |
|  | Lower limb FMA | 1w | WMD=-1.78(-7.15,3.59) | \ | 0.52 | \ | Very Low |
|  |  | 4w | WMD=-1.33(-7.98,5.32) | \ | 0.69 | \ | Very Low |
|  | Lower limb ROM | Immediate | WMD=2.43(-0.45,5.31) | \ | 0.10 | 0.0% | Low |
|  |  | 1w | WMD=1.47(-3.02,5.96) | \ | 0.52 | 59.0% | Very Low |
|  |  | 3-4w | WMD=3.26(0.25,6.28) | \ | 0.03 | 3.0% | Low |
|  |  | 12w | WMD=10.50(3.09,17.91) | \ | 0.005 | \ | Very Low |
|  | TUG | Immediate | WMD=-3.72(-5.64,-1.80) | \ | 0.0001 | \ | Very Low |
|  |  | 1w | WMD=-5.65(-7.89,-3.41) | \ | <0.00001 | 0.0% | Low |
|  |  | 3-4w | WMD=-2.25(-4.57,0.06) | \ | 0.06 | 0.0% | Very Low |
|  |  | 12w | WMD=-5.80(-14.81,3.21) | \ | 0.21 | \ | Very Low |
| E6 Mihai EE et al. 2020 | MAS | Short-term | SMD=0.75(0.40,1.10) | Moderate | <0.0001 | 0.0% | Moderate |
|  |  | Long-term (3-12 w) | SMD=1.34(1.01,1.66) | Large | <0.00001 | 26.0% | Low |
|  |  | between-group comparison (Long-term) | SMD=0.32(-0.01,0.65) | Small | 0.06 | 0.0% | Low |
|  | MTS | Long-term (3-12 w) | SMD=0.56(0.01,1.22) | Moderate | 0.05 | 0.0% | Low |
|  | H/M | Immediate | SMD=0.06(-0.31,0.43) | Small | 0.75 | 0.0% | Low |
|  | VAS | Long-term (3-12 w) | SMD=0.35(-0.21,0.91) | Small | 0.23 | 0.0% | Low |
|  | PROM | Long-term (3-12 w) | SMD=0.69(0.20,1.19) | Moderate | 0.006 | 0.0% | Moderate |
|  | TUG | Immediate | SMD=-0.16(-0.22,0.53) | Small | 0.41 | 0.0% | Low |
| E7 Cabanas-Valdés R et al.2020 | Upper limb MAS | 24h | MD=-1.78(-2.02,-1.53) | \ | <0.001 | 98.0% | Low |
|  |  | 24h-3w | MD=-0.79(-0.99,-0.59) | \ | <0.00001 | 96.0% | Low |
|  |  | 4-12w | MD=-0.74(-1.22,-0.25) | \ | 0.003 | 99.0% | Low |
|  |  | >12w | MD=-0.53(-0.95,-0.11) | \ | 0.01 | 100.0% | Low |
|  | Upper limb FMA | 24h-3w | MD=0.94(0.42,1.47) | \ | 0.0004 | 98.0% | Low |
|  |  | 4-12w | MD=0.97(0.19,1.74) | \ | 0.01 | 99.0% | Low |
|  |  | >12w | MD=0.92(0.27,1.57) | \ | <0.006 | 99.0% | Low |
|  | VAS | 24h | MD=-1.71(-2.27,-1.04) | \ | <0.001 | 81.0% | Low |
|  |  | 24h-3w | MD=-1.56(-2.05,-1.07) | \ | <0.001 | 14.0% | Moderate |
| E8 Cabanas-Valdés R et al.2020 | Lower Limb MAS | Short-term | MD=0.48（0.10,0.85） | \ | 0.01 | 0.0% | Moderate |
|  |  | Mid-term | MD=0.77 (0.18, 1.36.) | \ | 0.01 | \ | Very Low |
|  |  | Long-term | MD=0.66（0.06,1.26） | \ | 0.03 | 0.0% | Moderate |
|  | ROM | Short-term | MD=1.37(0.20,3.82) | \ | 0.25 | 0.0% | Moderate |
|  |  | Mid-term | MD=3.11(-0.25,8.72) | \ | 0.28 | \ | Very Low |
|  |  | Long-term | MD=3.43（-3.14,10.00） | \ | 0.31 | 7.0% | Low |
|  | Lower Limb Function | Mid-term | SMD=0.32(-0.28,0.92) | Small | 0.30 | 0.0% | Very Low |
|  |  | Long-term | SMD=0.36(-0.24,0.97) | Small | 0.24 | 0.0% | Very Low |
| E9 Ou-Yang L et al.2023 | MAS | Short-term (2 w) | MD=-0.43(-0.77,-0.10) | \ | <0.01 | 95.0% | Low |
|  |  | Mid-term (2w-4w) | MD=-0.50(-0.81,-0.20) | \ | <0.01 | 82.0% | Low |
|  |  | Long-term (>4w and ≤3m) | MD=-0.81(-1.15,-0.47) | \ | <0.01 | 94.0% | Low |
|  | MTS | Short-term (<2 w) | MD=5.56(-0.95,12.07) | \ | <0.05 | 0.0% | Low |
|  |  | Mid-term (>2w and ≤4w) | MD=9.63(1.89,17.37) | \ | <0.05 | 14.0% | Low |
|  | FMA | Short-term (2 w) | MD=1.12(0.42,1.83) | \ | <0.01 | 86.0% | Low |
|  |  | Mid-term (>2w and ≤4w) | MD=0.01(-0.46,0.48) | \ | <0.01 | 86.0% | Very Low |
|  |  | Long-term (>4w and ≤3m) | MD=0.29(-0.46,1.04) | \ | <0.01 | 99.0% | Very Low |
| E10 Ke M et al.2024 | Lower limb MAS | \ | MD=-0.32(-0.41,-0.23) | \ | <0.00001 | 37.0% | Moderate |
|  | Lower limb FMA | \ | MD=2.05(1.32,2.78) | \ | <0.00001 | 8.0% | Moderate |
|  | Lower limb ROM | \ | MD=1.69(0.52,2.86) | \ | <0.05 | 26.0% | Moderate |
| E11 Li C et al.2024 | Lower limb MAS | \ | MD=-0.48(-0.58,-0.38) | \ | <0.001 | 0.0% | Moderate |
|  | FMA-LE | \ | MD=4.19(2.13,6.25) | \ | <0.001 | 88.0% | Very Low |
|  | Lower limb CSS | \ | MD=-1.84(-2.61,-1.07) | \ | <0.001 | 67.0% | Very Low |
|  | Lower limb PROM | \ | MD=3.9(2.04,5.14) | \ | <0.001 | 21.0% | Moderate |
| E12 Teng H et al.2024 | Lower limb MAS | \ | MD=-0.54(-0.71,-0.37) | \ | <0.00001 | 0.0% | Moderate |
|  | FMA-LE | \ | MD=2.48(1.53,3.44) | \ | <0.00001 | 0.0% | Moderate |
|  | Lower limb PROM | \ | MD=4.47(2.26,6.68) | \ | <0.0001 | 0.0% | Moderate |
|  | Lower limb VAS | \ | MD=-0.46(-1.52,0.59) | \ | 0.39 | 82.0% | Very Low |
| E13 Chen J et al.2024 | MAS | \ | MD=-0.32(-0.58,-0.06) | \ | 0.02 | 87.0% | Very Low |
|  | FMA | \ | MD=4.63(2.60,6.65) | \ | <0.001 | 71.0% | Very Low |
|  | MBI | \ | MD=8.21(3.91,12.50) | \ | <0.001 | 89.0% | Very Low |
|  | PROM | \ | MD=5.88(1.33,10.42) | \ | 0.01 | 77.0% | Very Low |
| E14 Afzal B et al.2024 | MAS | Short-term ≤3w | MD=0.260(-0.058,0.578) | \ | 0.109 | 0.0% | Low |
|  |  | Long-term | MD=0.626 (0.133, 1.119) | \ | 0.013 | 0.0% | Moderate |
|  | ROM | Short-term ≤3w | MD=0.604(-0.234,0.973) | \ | 0.001 | 0.0% | Low |
|  |  | Long-term | MD=0.573(0.074,1.072) | \ | 0.02 | 13.0% | Moderate |
|  | Lower limb function | Short-term ≤3 w | MD=0.722(0.231,1.214) | \ | 0.004 | 0.0% | Moderate |
|  |  | Long-term>3w | MD=0.866(0.240,1.492) | \ | 0.007 | 0.0% | Moderate |
|  | TUG | \ | MD=0.174(-0.151,0.499) | \ | 0.294 | 0.0% | Very Low |
|  | 10MWT | Long-term >3w | MD=0.543(-0.307,1.394) | \ | 0.21 | 77.0% | Very Low |
|  | BI | Long-term>3w | MD=0.660(0.164,1.156) | \ | 0.009 | 0.0% | Low |
| E15 Liu W et al.2024 | MAS | Upper limb | MD=0.28(0.06,0.50) | \ | 0.01 | 38.0% | Low |
|  |  | Lower limb | MD=0.33(0.06,0.66) | \ | 0.02 | 0.0% | Moderate |
|  | VAS | Upper limb | MD=0.82(0.03,1.60) | \ | 0.04 | 54.0% | Low |
|  | PROM | Upper limb | MD=5.87(-4.36,16.10) | \ | 0.26 | 70.0% | Very Low |
|  |  | Lower limb | MD=4.33(2.18,6.48) | \ | <0.0001 | 0.0% | Moderate |
|  | FMA | Upper limb | MD=4.31(2.89,5.73) | \ | <0.00001 | 0.0% | Moderate |
|  |  | Lower limb | MD=2.58(0.97,4.18) | \ | 0.002 | 0.0% | Moderate |
| E16 Zhao H et al.2025 | Lower limb MAS (post-treatment) | \ | MD=-0.46(-0.66,-0.27) | \ | <0.001 | 56.0% | Low |
|  | Lower limb MAS (follow-up) | \ | MD=-0.53(-0.72,-0.34) | \ | <0.001 | 0.0% | Moderate |
|  | Lower limb FMA | \ | MD=4.95(2.42,7.48) | \ | <0.001 | 91.0% | Very Low |
|  | PROM | \ | MD=2.49(1.41,3.57) | \ | <0.0001 | 43.0% | Low |
|  | TUG | \ | MD=-5.48(-9.54,-1.42) | \ | 0.008 | 34.0% | Low |
|  | H/M | \ | MD=-0.34(-1.34,0.66) | \ | 0.50 | 99.0% | Very Low |
| E17 Sun J et al.2025 | MAS | Short-term <4w | MD=-0.75(-1.14,-0.36) | \ | <0.001 | 69.0% | Low |
|  |  | Long-term ≥4w | MD=-1.15(-1.14,-0.88) | \ | <0.001 | 30.0% | Moderate |
|  |  | Upper limb | MD=-0.77(-1.08,-0.46) | \ | <0.001 | 66.0% | Low |
|  |  | Lower limb | MD=-1.23(-1.64,-0.82) | \ | <0.001 | 0.0% | Moderate |

|  |
| --- |

**
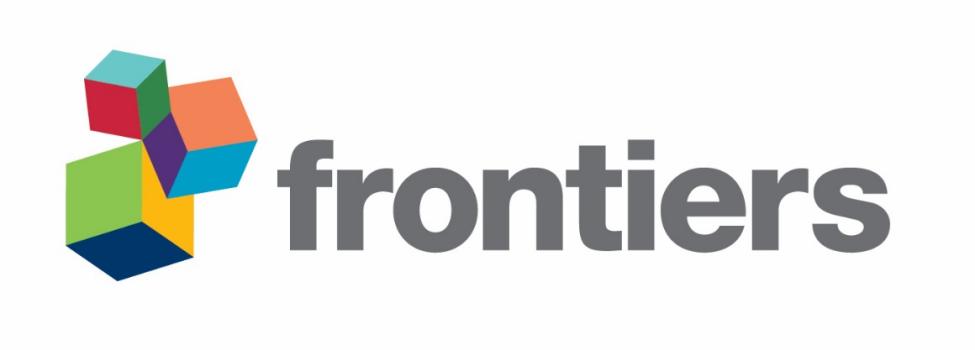
**
